# Quality of Information Regarding Repair Restorations on Dentist Websites: Systematic Search and Analysis

**DOI:** 10.2196/17250

**Published:** 2020-04-15

**Authors:** Philipp Kanzow, Amelie Friederike Büttcher, Annette Wiegand, Falk Schwendicke

**Affiliations:** 1 Department of Preventive Dentistry, Periodontology and Cariology University Medical Center Göttingen Göttingen Germany; 2 Division of Medical Education Research and Curriculum Development University Medical Center Göttingen Göttingen Germany; 3 Department of Operative and Preventive Dentistry Charité – Universitätsmedizin Berlin Berlin Germany

**Keywords:** evidence-based dentistry, internet, restoration repair, shared decision making

## Abstract

**Background:**

Repairing instead of replacing partially defective dental restorations represents a minimally invasive treatment concept, and repairs are associated with advantages over complete restoration replacement. To participate in the shared decision-making process when facing partially defective restorations, patients need to be aware of the indications, limitations, and advantages or disadvantages of repairs. Patients are increasingly using the internet to gain health information like this online.

**Objective:**

We aimed to assess the quality of German-speaking dentist websites on repairs of partially defective restorations.

**Methods:**

Three electronic search engines were used to identify German-speaking websites of dental practices mentioning repairs. Regarding information on repairs, websites were assessed for (1) technical and functional aspects, (2) comprehensiveness of information, and (3) generic quality and risk of bias. Domains 1 and 3 were scored using validated tools (LIDA and DISCERN). Comprehensiveness was assessed using a criterion checklist related to evidence, advantages and disadvantages, restorations and defects suitable for repairs, and information regarding technical implementation. Generalized linear modeling was used to assess the impact of practice-specific parameters (practice location, practice setting, dental society membership, and year of examination or license to practice dentistry) on the quality of information. An overall quality score was calculated by averaging the quality scores of all three domains and used as primary outcome parameter. Quality scores of all three domains were also assessed individually and used as secondary outcomes.

**Results:**

Fifty websites were included. The median score of quality of information was 23.2% (interquartile range [IQR] 21.7%-26.2%). Technical and functional aspects (55.2% [IQR 51.7%-58.6%]) showed significantly higher quality than comprehensiveness of information (8.3% [IQR 8.3%-16.7%]) and generic quality and risk of bias (3.6% [IQR 0.0%-7.1%]; *P*<.001/Wilcoxon). Quality scores were not related to practice-specific parameters (*P*>.05/generalized linear modeling).

**Conclusions:**

The quality of German-speaking dentist websites on repairs was limited. Despite sufficient technical and functional quality, the provided information was neither comprehensive nor trustworthy. There is great need to improve the quality of information to fully and reliably inform patients, thereby allowing shared decision making.

## Introduction

Repairs of partially defective restorations represent a minimally invasive treatment concept and are associated with a number of advantages over complete restoration replacement. In recent years, numerous studies have focused on the repair behavior of dentists, dental students, and dental educators [1,2]. Both retrospective [3-6] and prospective [7] clinical studies as well as a wide range of repair protocols based on numerous in vitro studies [8] are available, and even economic evaluations [9] have been published. The acceptance of dentists, and presumably also patients, toward repairs can be regarded as high, with patient acceptance having been reported to range from 89% to 93% (these numbers are based on interviewing dentists, however) [10-13].

To allow patients to participate in the shared decision-making process when facing partially defective restorations, both patients and dentists need to be aware of the indications, limitations, and advantages or disadvantages of repairs. For patients, such information will often come from their dentist (eg, during a consultation). Increasingly, however, patients may actively assess information like this online (eg, on their dentist’s website) [14,15]. Besides information, patients might also look online for a dentist able to deliver the requested care. Ideally, the information provided on dentists’ websites regarding treatments (like repairs) should be unbiased and comprehensive, allowing patients to come to an informed decision instead of being misinformed or biased. Until now, whether dentists’ websites allow patients to gain such comprehensive and trustworthy information on restoration repair has not been assessed.

Therefore, this study aimed to assess the quality of German-speaking dentists’ websites presenting information on repair restorations across three domains: (1) technical and functional aspects, (2) comprehensiveness of repair-specific information, and (3) generic quality and risk of bias. The null hypothesis was that practice-specific parameters do not impact website information quality.

## Methods

The reporting of this study follows the Preferred Reporting Items for Systematic Reviews and Meta-Analyses (PRISMA) and the Enhancing Transparency in Reporting the Synthesis of Qualitative Research (ENTREQ) statements [16,17].

### Search Strategy

Three electronic search engines (google.de, bing.de/yahoo.de, ask.com) were used. Searches were performed on April 28 and 29, 2019, using different search strategies, as google.de offers only limited options to combine multiple search terms with Boolean operators. Search terms represent different combinations of the German words for repair restoration(s), composite(s), and dentist(s) (Multimedia Appendix 1). A computer running macOS 10.14.4 (Apple Inc) connected to the internet in Germany was used. Cookies and browser history of Firefox Quantum 66.0.3 (Mozilla Foundation) were cleared and the default setting of each search engine was used.

In total, 2864 webpages were displayed as “most relevant” sites (google.de: 1299, bing.de/yahoo.de: 1295, ask.com: 270), and the search was not expanded beyond this number of displayed webpages assuming saturation. Also, advertisements (ie, websites from page owners paying a fee to have their website prominently displayed) were not additionally assessed.

For the purpose of this study, only websites from dentists were included. Patients are likely to be looking at these sources only while searching for a person able to deliver appropriate dental care. Therefore, websites from or associated with dental laboratories or supply and materials companies, forums and blogs operated by nondentists, dental regulatory bodies, dental schools and clinics, research agencies, or otherwise public bodies were excluded. Notably, however, patients may well find these informative, too.

The remaining 820 webpages were screened in full text. Webpages containing irrelevant information were excluded, leaving 74 webpages that were potentially eligible. Finally, after removal of duplicates, 50 websites fulfilled the inclusion criteria: (1) page freely accessible, (2) German language, (3) posted by a dental practice or practice cooperation, (4) mentions repairs. Websites containing multiple eligible webpages (ie, published under the same domain or published from the same practice) were jointly assessed as one website. The full search workflow is shown in Multimedia Appendix 2.

### Data Extraction

The following parameters were collected from the websites, if available: (1) practice name, (2) URL, (3) country, (4) practice location (rural, town [<100,000 inhabitants], or city [≥100,000 inhabitants]), (5) practice setting (single practitioner, multiple dentists, or practice cooperation), (6) dentist’s gender (female, male, mixed [in case of multiple dentists or practice cooperation]), (7) dental society memberships, (8) year of examination or approbation, and (9) information regarding repairs (Multimedia Appendix 3). Information regarding dental society memberships and year of examination or approbation were cross-referenced from dental societies’ member information pages (ie, German Society of Dentistry and Oral Medicine [DGZMK], Swiss Dental Association [SSO]) or curriculum vitae published elsewhere (ie, in dentists’ dissertations and public profiles at the social networking sites XING or LinkedIn), if information was not already listed on dentist websites. In case of multiple dentists or practice cooperations, the average years of examination or approbation was used.

### Outcomes

Website quality regarding information on repairs was systematically assessed across three different domains: (1) technical and functional aspects (Table 1), (2) comprehensiveness of information (Table 2), and (3) generic quality and risk of bias (Table 3). Assessment was independently performed by two authors (PK, AFB). Discrepancies were resolved through discussion.

The established and validated LIDA instrument (version 1.2) [18] was used to assess items in domain 1 and DISCERN instrument [19] was used in domain 3. In dentistry, such tools have been successfully applied to evaluate the quality of information on websites regarding dental caries [20,21], periodontitis [22], root canal treatment versus implant placement [23], and orthodontics [24-29]. For this study, both LIDA and DISCERN have been slightly modified to uniformly score all domains on an ordinal scale as 0 (never or no), 1 (sometimes or partially), and 2 (mostly, always, or yes).

To assess items in domain 2, a structured checklist with 6 subdomains was developed by the authors focusing on the evidence (2.1); advantages (2.2) and disadvantages (2.3) of repair restorations; restorations (2.4) and defects (2.5) suitable for repairs; and technical implementation of repairs (2.6). The same 3-point ordinal scale was used. As the number of items within each domain differed, an overall quality score was calculated by averaging the quality scores (relative percentages) of all three domains, assuming them to be of equivalent importance. This score was used as primary outcome parameter. Quality scores (relative percentages) of all three domains were also assessed individually and used as secondary outcomes.

**Table 1:** Subdomains regarding technical and functional aspects (domain 1) were assessed using the modified LIDA instrument (version 1.2) [18].

Subdomain and item		Median (IQR^a^; min-max^b^)	
**1.1 Accessibility**			
	Does it work on a range of browsers?^c^		2 (2-2; 1-2)
	Is the information available full text without registration, log-in or subscription?		2 (2-2; 2-2)
**1.2 Usability**			
	Is there a clear statement of who this website is for?		2 (2-2; 0-2)
	Is the level of detail appropriate to their^d^ level of knowledge?		0 (0-0; 0-2)
	Is the layout of the main block of information clear and readable?		2 (1-2; 1-2)
	Is the navigation clear and well structured?		2 (2-2; 1-2)
	Can you always tell your current location in the site?		2 (1-2; 0-2)
	Is the colour scheme appropriate and engaging?		1.5 (1-2; 0-2)
	Is the same page layout used throughout the site?		2 (2-2; 2-2)
	Do navigational links have a consistent function?^e^		1 (1-2; 0-2)
	Is the site structure (categories or organisation of pages) applied consistently?		2 (1-2; 0-2)
	Does the site provide an effective search function?^f^		0 (0-0; 0-1)
	Does the site provide effective browsing facilities?		2 (1-2; 0-2)
	Does the design minimize the cognitive overhead?		1 (1-2; 0-2)
	Does the site support the normal browser navigational tools?		2 (2-2; 2-2)
	Can you use the site without third party plugins?		2 (2-2; 2-2)
	Can the user make an effective judgment of whether the site applies to them?		2 (2-2; 1-2)
	Is the website interactive?		1 (0-1; 0-2)
	Can the user personalise their experience of using the site?		0 (0-0; 0-2)
	Does the website integrate nontextual media?		0 (0-0; 0-2)
**1.3 Reliability**			
	Does the site respond to recent events?		0 (0-1; 0-2)
	Can users submit comments on specific content?		0 (0-0; 0-2)
	Is site content updated at an appropriate interval?		0 (0-1; 0-2)
	Is it clear who runs the site?		2 (2-2; 2-2)
	Is it clear who pays for the site?		0 (0-0; 0-2)
	Is there a declaration of the objectives of the people who run the site?		2 (1-2; 0-2)
	Does the site report a clear content production method?		0 (0-0; 0-2)
	Is this a robust method?		0 (0-0; 0-1)
	Can the information be checked from original sources?		0 (0-0; 0-2)

^a^IQR: interquartile range.

^b^min-max: minimum and maximum score.

^c^Apple Safari 12.1, Firefox Quantum 66.0.3 for Mac, Google Chrome 74.0.3729 for Mac, and Microsoft Internet Explorer 11 were tested.

^d^The patients.

^e^As part of this item, websites were screened for broken links using a free online tool [30].

^f^Search terms “repariert,” “reparieren,” “Reparatur,” “Reparaturen,” “reparaturfähig,” “Reparaturfähigkeit,” “Füllungsreparatur,” “Füllungsreparaturen,” “Füllungserweiterung,” “Füllungserweiterungen,” “Reparaturfüllung,” “Reparaturfüllungen” were tested.

**Table 2:** Subdomains regarding treatment-related aspects (domain 2) were assessed.

Subdomain and item		Median (IQR^a^; min-max^b^)	
**2.1 Evidence of repair restorations**		0 (0-0; 0-2)	
	Are success rates or annual failure rates of repairs and replacements listed?		
	Are guidelines or scientific recommendations discussed?		
	Is literature cited?		
**2.2 Advantages of repair restorations**		0 (0-0; 0-2)	
	Is preservation of tooth substance (less invasive, less traumatic) mentioned?		
	Is risk reduction of treatment-related complications (eg, potentially harmful effects to the pulp or iatrogenic damage of neighboring teeth) discussed?		
	Are reduced costs mentioned?		
	Is reduced treatment time mentioned?		
**2.3 Disadvantages of repair restorations**		0 (0-0; 0-0)	
	Are disadvantages of repair restorations discussed?		
**2.4 Restorations suitable for repair**		1 (1-1; 0-2)	
	Are amalgam restorations mentioned?		
	Are composite restorations mentioned?		
	Are ceramic restorations mentioned?		
	Are full-metal restorations mentioned?		
	Are further indirect restorations mentioned?		
**2.5 Defects suitable for repair**		0 (0-0.75; 0-2)	
	Is damage, fracture, partial loss, or partial defect of restoration discussed?		
	Is secondary caries mentioned?		
	Is (marginal) discoloration mentioned?		
	Is ceramic chipping mentioned?		
	Are marginal defects or gaps mentioned?		
**2.6 Technical implementation of repair**		0 (0-0; 0-2)	
	Is sandblasting mentioned?		
	Is application of silane or universal primers mentioned?		
	Are repair materials mentioned?		

^a^IQR: interquartile range.

^b^min-max: minimum and maximum score.

**Table 3:** Subdomains regarding generic quality and risk of bias (domain 3) were assessed using the modified DISCERN instrument [19].

Subdomain and item		Median (IQR^a^; min-max^b^)
**3.1 Reliability**		
	Are the aims clear?	0 (0-0; 0-0)
	Is it^c^ relevant?	0 (0-1; 0-2)
	Is it clear what sources of information were used to compile the publication?	0 (0-0; 0-2)
	Is it clear when the information used or reported in the publication was produced?	0 (0-1; 0-2)
	Is it^c^ balanced and unbiased?	0 (0-0; 0-0)
	Does it^c^ provide details of additional sources of support and information?	0 (0-0; 0-0)
	Does it^c^ refer to areas of uncertainty?	0 (0-0; 0-0)
**3.2 Quality**		
	Does it^c^ describe how each treatment works?	0 (0-0; 0-2)
	Does it^c^ describe the benefits of each treatment?	0 (0-0; 0-2)
	Does it^c^ describe the risks of each treatment?	0 (0-0; 0-0)
	Does it^c^ describe what would happen if no treatment is used?	0 (0-0; 0-0)
	Does it^c^ describe how the treatment choices affect overall quality of life?	0 (0-0; 0-0)
	Is it clear that there may be more than one possible treatment choice?	0 (0-0; 0-0)
	Does it^c^ provide support for shared decision making?	0 (0-0; 0-0)

^a^IQR: interquartile range.

^b^min-max: minimum and maximum score.

^c^Websites’ content.

### Statistical Analysis

For each domain, a quality score (relative percentage: website score on all of the respective items divided by the maximum possible score sum) was calculated. Furthermore, an averaged overall quality score based on all three domains was calculated. As data were not normally distributed according to the Shapiro-Wilk test, descriptive statistical analysis contained median, quartiles, and ranges.

Differences in website scores between the three domains were analyzed using Wilcoxon signed-rank tests with a Bonferroni-Holm correction. Generalized linear modeling was used to assess the impact of practice-specific parameters on domain-related quality and the averaged overall quality score: (1) practice location (rural, town, or city); (2) practice setting (single practitioner, multiple dentists, or practice cooperation); (3) dental society membership (yes or no); and (4) year of examination or approbation. A multivariable analysis was performed and covariates entered simultaneously. Only main effects without interaction terms were tested. If no information regarding dental society membership was available, we scored this as no. If year of examination or approbation was not available, websites were treated as randomly missing and excluded from the regression analysis (n=4). Statistical analysis was performed using SPSS Statistics for Macintosh version 26.0.0.0 (IBM Corp). Statistical significance was set at *P*<.05.

## Results

In total, 50 websites fulfilled the inclusion criteria. Characteristics of the included websites are shown in Table 4 (full data of all included websites are shown in Multimedia Appendix 3). Briefly, the majority of websites were from practices in Germany and situated in towns or cities. Half of the practices had single practitioners, and about half of the dentists running the websites were members of dental societies.

**Figure 1:**
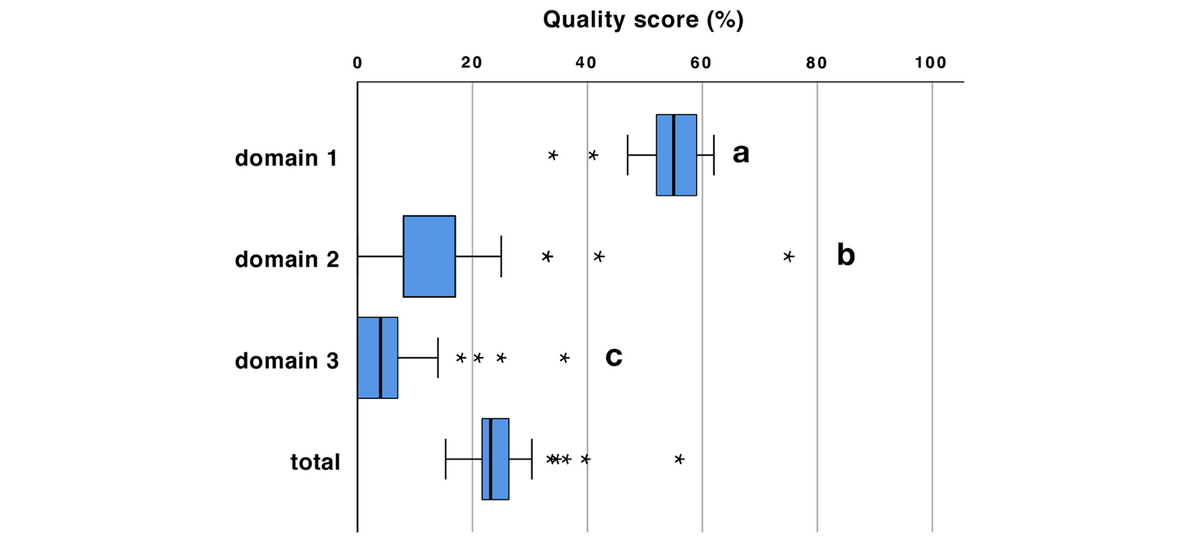
Quality of different domains (relative percentage of maximum possible score sum) and total score (averaging the results from all three domains). Domain 1: technical and functional aspects; domain 2: comprehensiveness of information; domain 3: generic quality and risk of bias. Significant differences between domains are marked by different letters (*P*<.001/Wilcoxon). Outliers are marked with an asterisk (*).

The median score for quality of information was 21.2% (interquartile range [IQR] 20.0%-22.3%) (Figure 1). Technical and functional aspects (55.2% [IQR 51.7%-58.6%]) showed significantly higher quality than did comprehensiveness of information (8.3% [IQR 8.3%-16.7%]) and generic quality and risk of bias (3.6% [IQR 0.0%-7.1%]; *P*<.001/Wilcoxon, Tables 1-3).

**Table 4:** Practice-specific parameters of the included websites (n=50).

Variable and attribute		Value
**Country, n (%)**		
	Germany	38 (76)
	Switzerland	7 (14)
	Austria	4 (8)
	Hungary	1 (2)
**Practice location, n (%)**		
	Rural	10 (20)
	Town	17 (34)
	City	23 (46)
**Practice setting, n (%)**		
	Single practitioner	25 (50)
	Multiple dentists or practice cooperation	25 (50)
**Gender, n (%)**		
	Female	8 (16)
	Male	16 (32)
	Mixed	26 (52)
**Dental society membership, n (%)**		
	No	24 (48)
	Yes	26 (52)
Year of examination or approbation^a,^^b^, mean (SD)		1996 (9)

^a^In case of multiple dentists or practice cooperations, the years of examination or approbation were averaged, if available.

^b^There were 4 missing values.

Within multivariable regression analysis, none of the practice-specific parameters had a significant impact on the averaged overall quality score or domain-related quality scores (*P*>.05/generalized linear modeling, Table 5).

**Table 5:** Association between practice-specific parameters and website quality.

Outcome	Model fit		Practice location^a^		Practice setting (ref. single practitioner)^a^	Dental society membership (ref. no or unknown)^a^	Year of examination or approbation^a,b^
	Likelihood	*P* value	Towns (ref. rural)	Cities (ref. rural)			
Domain 1	7.88	.16	–0.45 (–4.82 to 3.93)	2.58 (–1.68 to 6.83)	1.09 (–1.87 to 4.05)	1.38 (–1.81 to 4.56)	0.12 (–0.05 to 0.29)
Domain 2	5.08	.41	6.57 (–4.69 to 17.84)	1.87 (–9.08 to 12.82)	4.51 (–3.11 to 12.13)	2.54 (–5.65 to 10.74)	–0.28 (–0.72 to 0.16)
Domain 3	3.09	.69	3.86 (–2.64 to 10.36)	2.08 (–4.23 to 8.40)	1.86 (–2.54 to 6.26)	1.18 (–3.55 to 5.91)	–0.11 (–0.36 to 0.15)
Total	3.20	.67	3.33 (–2.61 to 9.27)	2.18 (–3.60 to 7.95)	2.49 (–1.53 to 6.50)	1.70 (–2.62 to 6.02)	–0.09 (–0.32 to 0.14)

^a^Regression coefficients with 95% confidence intervals are shown. ^b^In case of multiple dentists or practice cooperations, the years of examination or approbation were averaged, if available.

## Discussion

### Principal Findings

In the D-A-CH countries (Germany, Austria, Switzerland), about 90% of the population has access to the internet [31]. Information on health-related aspects is increasingly assessed online, often using search engines [14,15]. Due to the broad access to the internet, operating a website has become the standard for most companies and businesses including dentists.

### Search Strategy

Regarding dental health, information on dental practice websites is of special interest as patients are likely to access those websites while searching for information and an appropriate dentist. Therefore, our study focused on dentist websites only. Websites were identified using different search engines with a combined market share of more than 99% in Germany [32]. The search was performed using consumer search engines only as patients are unlikely to use scientific databases (eg, Medline).

### Information Regarding Repairs

We found that only a small number of dentists included information about repairs on their websites. Dentist websites showed sufficient quality regarding technical and functional aspects but were not seen as fully trustworthy (generic quality was low, and there was a high risk of bias present). Comprehensiveness of repair-specific information was also rated low. This is in line with previous studies assessing the quality of websites regarding different dental health-related information [20,22,23,25,26,28,29]. Dental health-related information was not comprehensive and of lower quality than websites' technical and functional aspects.

A number of reasons for these findings are conceivable. First, dentists might not have enough time to create and maintain a content-comprehensive website. Dentists might also feel that informing patients online is not necessary or that it is not their task to supply patients with comprehensive health care information on their websites. The perceived lack of financial gain from providing such content online may add to this. Also, provided online content might need to be discussed with patients at the next appointment, which may be seen as a waste of time. Data from the United States demonstrated that physicians perceive appointments as less efficient and more difficult if patients have already gained information online [33]. In contrast, insufficient knowledge regarding repairs among dentists is unlikely to be a reason, as repairs are frequently taught at dental schools in Germany and all over the world [1,2]. However, dentists might regard implantology or orthodontic information to be of more importance than information on repairs, resulting in higher quality scores concerning technical aspects and generic quality and risk of bias of these websites (also measured using LIDA and DISCERN) [23,27,29].

### Impact of Practice-Specific Parameters

We did not find any significant association between practice-specific parameters and website quality scores. We therefore must reject our hypothesis. This is a surprising outcome, as a range of parameters including those related to the individual practitioner and their practice seem to impact on repair behavior [1]. For example, low dentist density (ie, in a rural area), more experience or knowledge (ie, being a member of the dental associations), fewer years since dental school graduation, and working in larger group practices (ie, with multiple dentists) have been found to facilitate repairs. Also, we assumed website quality would be higher in younger dentists being more comfortable with technology and in larger group practices (with higher budgets for an online presence and marketing). Notably, a previous study on dentist websites and their quality also failed to demonstrate significant associations with most of such practice-specific parameters [22]. We mainly ascribe this to the fact that the overall quality was too poor throughout different websites, and dentists generally do not seem to prioritize providing information on repairs on their website regardless of their background or practice environment.

### Limitations

This study has a number of limitations. First, the relatively small number of included websites (n=50) must be noted. Notably, the sample size was not based on a formal sample size estimation but guided by a previous study [22] and the availability of websites. Our study might have been underpowered, and the lack of significant associations should hence be interpreted with caution. Second, we focused on German-speaking websites only. It is possible, albeit unlikely, that websites in other languages present a higher quality (eg, with regard to periodontitis, both German- and English-speaking websites showed a low quality of information) [22,34]. Last, we used established and validated criteria to assess technical aspects and generic quality and risk of bias but developed an assessment checklist for the repair-specific quality and comprehensiveness on our own. The validity of this checklist was not formally tested, and using another checklist may lead to different results.

### Overcoming Observed Shortcomings

To overcome the shortcomings of dentist websites, a number of interventions are conceivable. Regulatory and legislative bodies might enforce better information standards. Professional dental bodies might assist dentists by providing high-quality information suitable for adoption on dentists’ websites. Alternatively, dentists could provide links to other validated websites or organizations able to provide comprehensive information, such as dental research societies, thereby reducing the burden for the individual dentist to provide and maintain high-quality information. We did not check society websites as it can be assumed that information presented is both trustworthy and comprehensive.

### Conclusion

In conclusion, only a minority of dentist websites informed patients about repair restorations. Despite sufficient technical and functional quality, the websites that did mention repairs were not comprehensive and prone to a high risk of bias. Dentists are encouraged to provide better and more trustworthy health information, including but not limited to repairs. Professional or regulatory bodies might assist dentists by providing high-quality information suitable for adoption on dentist websites. In the meantime, patients must be aware of the limitations and should seek information regarding repairs elsewhere.

## Appendix

Multimedia Appendix 1 Search terms used in the research.

Multimedia Appendix 2 Flowchart representing the search workflow.

Multimedia Appendix 3 List of websites with information on repair restorations or restoration repair.

## Abbreviations

DGZMK: German Society of Dentistry and Oral Medicine
ENTREQ: Enhancing Transparency in Reporting the Synthesis of Qualitative Research
IQR: interquartile range
PRISMA: Preferred Reporting Items for Systematic Reviews and Meta-Analyses
SSO: Swiss Dental Association
