# Supplementary material for: Quality of Information Regarding Repair Restorations on Dentist Websites: Systematic Search and Analysis
Source: J Med Internet Res. 2020 Apr 15;22(4):e17250. doi: 10.2196/17250 (PMC7191344; doi:10.2196/17250)
Supplement: Multimedia Appendix 1 [file jmir_v22i4e17250_app1.doc]

Search terms used.

|  | **google.de** | **bing.de / yahoo.de, ask.com** |
| --- | --- | --- |
| # 1 | füllungsreparatur OR füllungsreparaturen OR füllungserweiterung OR füllungserweiterungen OR reparaturfüllung OR reparaturfüllungen OR füllungsreparatur OR füllungsreparaturen | füllungsreparatur OR füllungsreparaturen OR füllungserweiterung OR füllungserweiterungen OR reparaturfüllung OR reparaturfüllungen OR füllungsreparatur OR füllungsreparaturen |
| # 2 | repariert OR reparieren OR reparatur OR reparaturen OR reparaturfähigkeit OR reparaturfähig "füllung" | (repariert OR reparieren OR reparatur OR reparaturen OR reparaturfähigkeit OR reparaturfähig) AND füllung |
| # 3 | repariert OR reparieren OR reparatur OR reparaturen OR reparaturfähigkeit OR reparaturfähig "composit" "zahnarzt" | (repariert OR reparieren OR reparatur OR reparaturen OR reparaturfähigkeit OR reparaturfähig) AND composit AND zahnarzt |
| # 4 | repariert OR reparieren OR reparatur OR reparaturen OR reparaturfähigkeit OR reparaturfähig "komposit" "zahnarzt" | (repariert OR reparieren OR reparatur OR reparaturen OR reparaturfähigkeit OR reparaturfähig) AND komposit AND zahnarzt |
| # 5 | repariert OR reparieren OR reparatur OR reparaturen OR reparaturfähigkeit OR reparaturfähig "composit" "zahnärztin" | (repariert OR reparieren OR reparatur OR reparaturen OR reparaturfähigkeit OR reparaturfähig) AND composit AND zahnärztin |
| # 6 | repariert OR reparieren OR reparatur OR reparaturen OR reparaturfähigkeit OR reparaturfähig "komposit" "zahnärztin" | (repariert OR reparieren OR reparatur OR reparaturen OR reparaturfähigkeit OR reparaturfähig) AND komposit AND zahnärztin |
| # 7 | "reparatur von füllungen" | "reparatur von füllungen" |
| # 8 | "reparaturen von füllungen" | "reparaturen von füllungen" |

Search terms represent different combinations of the German words for “repair restoration(s)”, “composite(s)”, and “dentist(s)”.
