# Supplementary material for: Quality of Information Regarding Repair Restorations on Dentist Websites: Systematic Search and Analysis
Source: J Med Internet Res. 2020 Apr 15;22(4):e17250. doi: 10.2196/17250 (PMC7191344; doi:10.2196/17250)
Supplement: Multimedia Appendix 2 [file jmir_v22i4e17250_app2.pdf]

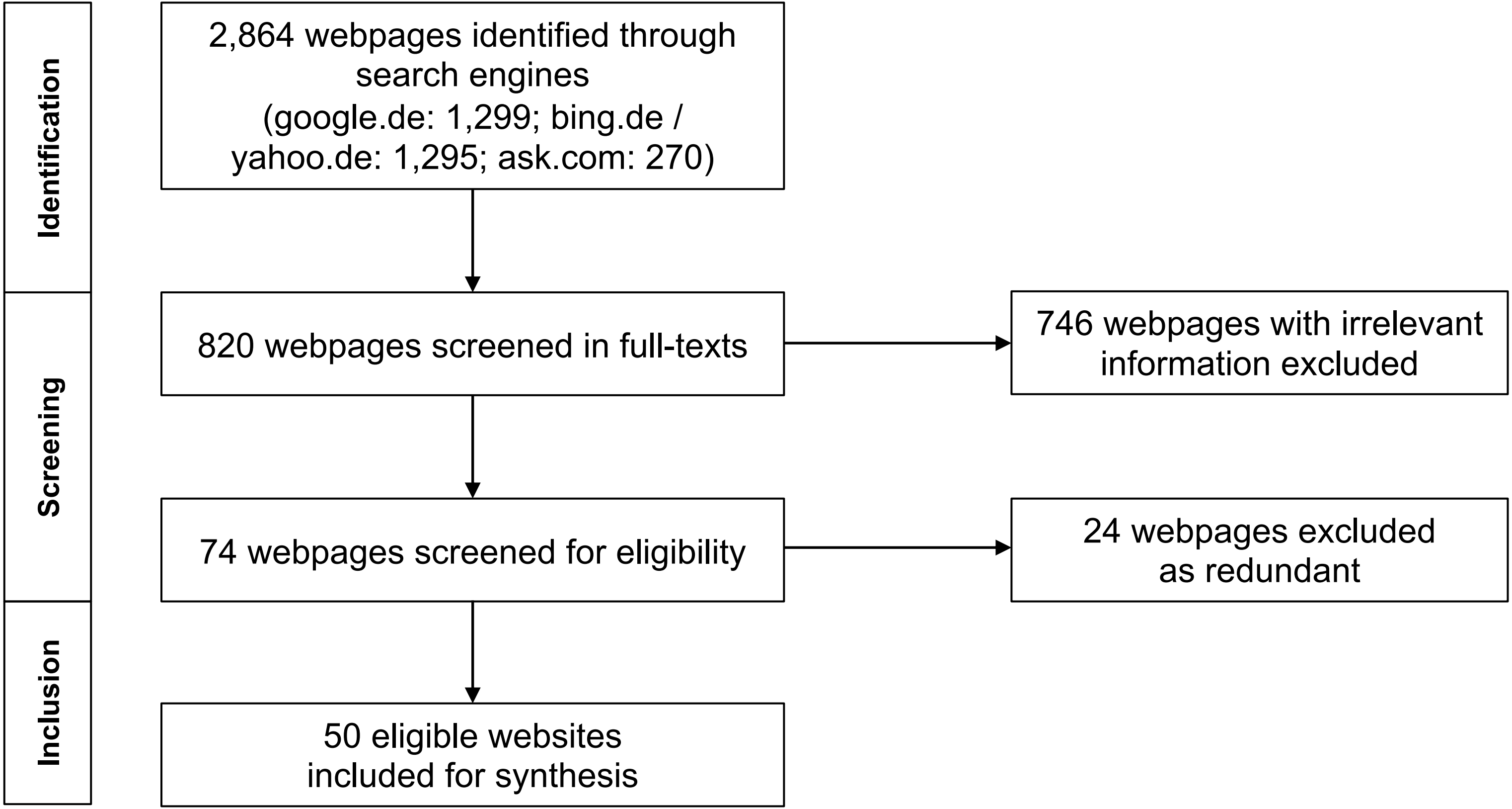
Identification
Screening
Inclusion
2,864 webpages identified through search engines (google.de: 1,299; bing.de / yahoo.de: 1,295; ask.com: 270)
820 webpages screened in full-texts
746 webpages with irrelevant information excluded
74 webpages screened for eligibility
24 webpages excluded as redundant
50 eligible websites included for synthesis
