# Supplementary material for: Quality of Information Regarding Repair Restorations on Dentist Websites: Systematic Search and Analysis
Source: J Med Internet Res. 2020 Apr 15;22(4):e17250. doi: 10.2196/17250 (PMC7191344; doi:10.2196/17250)
Supplement: Multimedia Appendix 3 [file jmir_v22i4e17250_app3.doc]

Included websites with information on repair restorations / restoration repair.

| **Practice** | **Uniform Resource Locator (URL)** | **Country** | **Practice location**  **[rural / town / city]** | **Practice setting**  **[single practitioner / multiple dentists]** | **Society membership** | **Year of examination / Approbation** | **Provided information on repairs** |
| --- | --- | --- | --- | --- | --- | --- | --- |
| Charles A. Smith, DDS & Kollegen | zahnarzt-aesthetik.de/leistungen/porzellan-veneers/ | DE | Heidelberg (city) | Single dentist (male) | *no information provided* | 1995 [1]* | - Restorations suitable for repairs (veneers) |
| Dr. Kremer | zahnarzt-freiburg-dr-kremer.de/fortbildung-zahnheilkunde.html | DE | Freiburg (city) | Single dentist (male) | *no information provided* | 1991 [2]** | - Restorations suitable for repairs (composite restorations) |
| Implanteer®, Dr. Hinze | implanteer.de/zahnaesthetik/zahnersatz-zahnaesthetik-muenchen/ | DE | Gräfelfing (rural) | Multiple dentists (one female, one male dentist) | BFSP, DGI, DGParo, DGZMK, EAED, EDA, EFP | 2005, 2015 | - Restorations suitable for repairs (composite restorations) - Defects suitable for repairs (damage, discoloration) |
| Gemeinschaftspraxis Dr. Schubert, Zweigardt | mikroskopzahnarzt.de  mikroskopzahnarzt.de/schöne-zähne/reparieren-oder-ersetzen/  mikroskopzahnarzt.de/praxis-aktuell/neues-aus-der-zahnmedizin/ | DE | Lauingen (town) | Multiple dentists (two male dentist) | *no information provided* | 1979, 1992 | - Evidence of repair restorations (success rates from cited literature [3], society recommendation) - Advantages of repair restorations (preserving tooth substance, reduced costs) - Restorations suitable for repairs (ceramic restorations, full-metal restorations, and composite restorations) - Defects suitable for repairs (fractures / partial loss of restorations, marginal defects, marginal discoloration) - Technical implementation of repairs (application of silane, repairs using composite) |
| Gemeinschaftspraxis für Zahnheilkunde, Dr. Stegner, Dr. Stegner | dres-stegner.de/praxisratgeber/direkte-fuellungstherapie | DE | Freystadt (town) | Multiple dentists (one female, one male dentist) | DGZMK, APW, DGI, DGZ, DGET, DGKiZ, DGZH, DGFDT***, DGPro*** | 1996, 1996 | - Restorations suitable for repairs (amalgam restorations, eventually composite restorations) - Technical implementation of repairs (repairs using amalgam or composite) |
| Praxis für Zahngesundheit, Dr. Kettler-Nölke | zahnarztpraxis-hamburg-niendorf.de/zahnfuellungen.php | DE | Hamburg (city) | Single practitioner (female) | DGZMK, DGCZ, DGI, DGZMK | 1973 [4]** | - Restorations suitable for repairs (composite restorations) |
| Privatzahnärztliche Praxis für Sanfte Zahnheilkunde | sanfte-zahnheilkunde.de/karies-heilen-ohne-bohrer/ | DE | Lindau (town) | Multiple dentists (two male dentists) | ADA, DGCZ, DGZ, DGZMK, DGParo | 1979, 2013 | - Advantages of repair restorations (preserving tooth substance) |
| Zahnärzte Buckow | zahnaerzte-buckow.de/aesthetik-berlin/zahnarzt-moderne-komposite.html | DE | Berlin (city) | Multiple dentists (two female, one male dentist) | DGET, DGZMK | 1994, 2010, 2015 | - Restorations suitable for repairs (composite restorations) - Defects suitable for repairs (discoloration) |
| Zahnärzte Dr. Linge, Dr. Gäbert-Gallo | zahnarzt-linge.de/leistungen/kompositefuellungen.html | DE | Kassel (city) | Multiple dentists (two female, one male dentist) | *no information provided* | *1973 [5]**, 2005 [6]**, 2007 [7]*** | - Restorations suitable for repairs (composite restorations) |
| Zahnärztin Dr. Schwarzer-Michalczik | zahnarzt-essen-heisingen.de/pages/202_fuellungen.php | DE | Essen (city) | Multiple dentists (one female, one male dentist) | *DGZMK, DGI, DGCZ***, DGMKG, APW, BDIZ, BDO* | *1984 [8]**, 1989 [9]*** | - Restorations suitable for repairs (ceramic inlays, composite restorations) - Defects suitable for repairs (secondary caries, marginal defects / gaps) |
| Zahnärztin Dr. Tilk | drtilk.de/schwerpunkte/moderne-füllungstherapie/ | DE | Nalbach (rural) | Multiple dentists (one female, one male dentist) | DGZMK | 2007, 2018 | - Restorations suitable for repairs (composite restorations) - Defects suitable for repairs (partial defect) |
| Zahnärztliche Gemeinschaftspraxis Dr Bettendorf, Dr. Mangelsdorf | zahnaerzte-tagesklinik.de/therapie/zahnersatz | DE | Hofheim (town) | Multiple dentists (two female, two male dentists) | DGZMK***, DGÄZ, DGFDT, DGI, DGParo | 1986, 1999, 2007, 2012 | - Restorations suitable for repairs (composite restorations) |
| Zahnärztliche Praxisgemeinschaft Dr. Leugner, Leugner | zahnarzt-bochum.blogspot.com/2009/02/reparaturen-nach-sturzen-durch-ein-neue.html  zahnarzt-herne.info/keramik.html | DE | Bochum (city) | Multiple dentists (one female, two male dentists) | DGZMK*** | 1987, 1997, 2008 [10]**** | - Advantages of repair restorations (reduced costs) - Restorations suitable for repairs (ceramic restorations) - Defects suitable for repairs (ceramic chipping) - Technical implementation of repairs (sandblasting) |
| Zahnarzt Dr. Huber | drpeterhuber.de/14-angebot/25-unser-behandlungss | DE | Kirchheim unter Teck (town) | Single practitioner (male) | AAP, DGÄZ, DGZMK, | 1985 | - Restorations suitable for repairs (ceramic restorations) |
| Zahnarzt Dr. Kuhmann | zahnnotizen.de/2011/01/10/defekte-kompositrestaurationen-reparieren-oder-ersetzen/ zahnnotizen.de/2016/03/24/reparatur-einer-amalgamfuellung-mit-komposit/  zahnnotizen.de/2011/11/22/reparatur-einer-vollkeramikkrone/  zahnnotizen.de/2010/10/07/komposit-zur-reparatur-eines-goldinlays/ | DE | Würzburg (city) | Single practitioner (male) | DGÄZ, DGI, DGKiZ, DGParo, DGZMK DNEbM | 1996 [11]** | - Evidence of repair restorations (cited literature [12]) - Advantages of repair restorations (reduced costs, reduced time given) - Restorations suitable for repairs (amalgam restorations, composite restorations, ceramic restorations, metal restorations) - Defects suitable for repairs (secondary caries, marginal gaps) |
| Zahnarzt Dr. Mundorf | dr-mundorf.de/behandlungen/minimalinvasive-fuellung/ | DE | Hamburg (city) | Single dentist (male) | *no information provided* | *1995 [13]*** | - Restorations suitable for repairs (composite restorations) |
| Zahnarzt Dr. Neumann | dr-neumann.info/leistungen_prophylaxe-praeventivzahnmedizin_cms.php | DE | Berlin (city) | Single practitioner (male) | AGET, APW, DGZ, DGZMK, VDZE, DGPro*** | 1994 | *none* |
| Zahnarzt Lorke | zahnarzt-lorke.de/leistungen/die-ersten-zaehne/fissurenversiegelung/ | DE | Würzburg (city) | Multiple dentists (two female, two male dentists) | DGZMK***, DGÄZ***, DGKiZ*** | *no information provided* | - Restorations suitable for repairs (fissure sealants) |
| Zahnarzt Schlamp | zahnarzt-schlamp.de/Composite%20Fuellungen.htm | DE | Cologne (city) | Single practitioner (male) | *no information provided* | *no information provided* | - Restorations suitable for repairs (composite restorations and all common restorative materials) - Technical implementation of repairs (repairs using composite) |
| Zahnarztpraxis Dakowitz | zahnarztpraxis-dakowitz.de/fuellungstherapie-zahnfuellungen-buchholz.html | DE | Buchholz (town) | Single practitioner (female) | *no information provided* | 1991 | - Advantages of repair restorations (preservation of tooth substance) - Restorations suitable for repairs (composite restorations) |
| Zahnarztpraxis Dr. Bauer | zahnarztbauer.de/behandlungen/konservierende-zahnheilkunde/ | DE | Heßdorf (rural) | Multiple dentists (one female, one male dentist) | DGI, DGZMK | 1998, *unknown* | *none* |
| Zahnarztpraxis Dr. Budzisch | dr-budzisch.de/fuellungen-inlays | DE | Berchtesgaden (town) | Multiple dentists (three male dentists) | *no information provided* | 1984, 1996, 2010 | - Restorations suitable for repairs (composite restorations) |
| Zahnarztpraxis Dr. Hanses | zahnarztpraxis-dr-hanses.de/herzlich_willkommen.htm | DE | Büren (town) | Single practitioner (female) | *no information provided* | 1985 | - Restorations suitable for repairs (composite restorations) |
| Zahnarztpraxis Dr. Hohmann | dr-hohmann.de/index.php?option=com_content&view=article&id=17&Itemid=37 | DE | Lollar (town) | Single dentist (male) | *no information provided* | *1985 [14]*** | - Advantages of repair restorations (reduced costs) - Restorations suitable for repairs (indirect restorations) - Defects suitable for repairs (ceramic chipping) |
| Zahnarztpraxis Dr. Hotz, Eckers | drhotzundeckers.de/leistungen/zahnerhaltung.html |  | Stuttgart (city) | Multiple dentists (two male dentists) | *no information provided* | 1988, 1988 | - Restorations suitable for repairs (composite restorations) - Technical implementation of repairs (repairs using composite) |
| Zahnarztpraxis Dr. Hout | dr-hout.de/fuellungeninlays/ | DE | Frankfurt (city) | Single dentist (male) | *no information provided* | 2001 | *none* |
| Zahnarztpraxis Dr. Leitenstorfer | dr-leitenstorfer.de/leistung/fuellungstherapie | DE | Munich (city) | Multiple dentists (two female dentists) | *no information provided* | 1992, 2010 | *none* |
| Zahnarztpraxis Dr. Rothballer | weisse-zaehne-amberg.de  weisse-zaehne-amberg.de/moderne-zahnerhaltung/zahnerhaltung-mit-compositen/  weisse-zaehne-amberg.de/moderne-zahnerhaltung/reparaturfreundlich/ | DE | Amberg (town) | Single practitioner (male) | *no information provided* | 1990 | - Restorations suitable for repairs (composite restorations) - Defects suitable for repairs (secondary caries) |
| Zahnarztpraxis Dr. Strohm | dr-strohm.de/leistungen/fuellungen-rekonstruktionen-aesthetische-korrekturen | DE | Tübingen (town) | Multiple dentists (one female, one male dentist) | *no information provided* | 1987, *unknown* | - Technical implementation of repairs (repairs using glass ionomer cement) |
| Zahnarztpraxis Dr. Schmidt, Dr. Stopperich | www.zahnarzt-in-koblenz.de/zahnerhalt/ | DE | Koblenz (city) | Multiple dentists (one female, one male dentist) | *no information provided* | *2002 [15]***, *unknown* | - Advantages of repair restorations (preservation of tooth substance) - Restorations suitable for repairs (composite restorations) - Defects suitable for repairs (secondary caries, marginal discoloration) - Technical implementation of repairs (sandblasting) |
| Zahnarztpraxis Dr. Torz | zahnarzt-torz.de/behandlungsspektrum/asthetische-zahnheilkunde/composite-fullung/ | DR | Geesthacht (town) | Single practitioner (male) | DGZI, DGZMK, SHGZMK*** | 1997 | - Restorations suitable for repairs (composite restorations) - Technical implementation of repairs (repairs using composite) |
| Zahnarztpraxis für Zahnerhaltung, Dr. Zantner | zahnarztpraxis-fuer-zahnerhaltung.de/Behandlung-Erwachsene-Zahnarzt.htm | DE | Munich (city) | Single practitioner (female) | DGZMK, DGZ | 1999 | - Advantages of repair restorations (preservation of tooth substance) |
| Zahnarztpraxis Krüger | zahnarzt-krueger-muenster.de/aesthetische-zahnmedizin/ | DE | Münster (city) | Single dentist (male) | *no information provided* | *2005* | - Restorations suitable for repairs (composite restorations) |
| Zahnarztpraxis Niedernjesa, Dr. Porschen, Dr. Kettler | zahn2500.com | DE | Niedernjesa (rural) | Multiple dentists (one female, one male dentist) | DGCZ, DGI, DGKiZ, DGParo, DGSZ, DGZMK | 1991 [16]**, *unknown* | - Advantages of repair restorations (reduced costs) - Restorations suitable for repairs (indirect restorations) |
| Zahnarztpraxis Saupe | zahnärzte-seehof.de/zahnluecke-fuellen/  zahnärzte-seehof.de/zahnumformung-und-lueckenschluss/ | DE | Teltow (town) | Multiple dentists (two male dentists) | *no information provided* | 2011, 2011 | - Restorations suitable for repairs (composite restorations) |
| Zahnarztpraxis Schimank | prenzlauer-berg-berlin-zahnarzt.de | DE | Berlin (city) | Single practitioner (male) | *no information provided* | 2012 | - Restorations suitable for repairs (composite restorations) |
| Zahnheilkunde im Stilwerk | dr-meynberg.de/zahnfuellungen | DE | Hamburg (city) | Multiple dentists (two female dentists) | *no information provided* | 1997, 2014 | - Restorations suitable for repairs (composite inlays) |
| Zahnheilkunde Oppermann | zahnheilkunde-oppermann.de/pages/aesthetik/zahnfarbene-fuellungen.php  zahnheilkunde-oppermann.de/pages/funktion/fuellungstherapie.php | DE | Dresden (city) | Single practitioner (male) | DGÄZ, DGZMK | *no information provided* | - Restorations suitable for repairs (composite restorations) |
| Aare Zahnarztpraxis Thun | thunerzahnarzt.ch/kunststoffe-komposite/ | CH | Thun (town) | Single practitioner (male) | SSO | 1999 | - Evidence of repair restorations (refers to society recommendations) - Restorations suitable for repairs (composite restorations) |
| Dr. Weilenmann | zahnarztweilenmann.ch/?Ordner=Leistung&SO=Reparaturen  zahnarztweilenmann.ch/?Ordner=Leistung&SO=Klebebruecken  zahnarztweilenmann.ch/?Ordner=Leistung&SO=Kronen | CH | Wetzikon (town) | Single practitioner (male) | SSGS, SSO | 1979 | - Restorations suitable for repairs (composite restorations, indirect restorations) - Defects suitable for repairs (fractures, secondary caries, ceramic chipping, missing proximal contact) - Technical implementation of repairs (repairs using composite) |
| Zahnärztliche Familienpraxis Dr. Recoder | familien-zahnarzt-baar.ch/leistungen/schne-zhne/kunststoff-fllungen | CH | Baar (rural) | Multiple dentists (two female dentists) | SSO, SVK | 2010, 2010 | - Restorations suitable for repairs (composite restorations) |
| Zahnarzt Gysin | beissfest.ch/kompetenzen/füllungen | CH | Sissach (rural) | Multiple dentists (one female, one male dentist) | SSO | 1994, 2018 | - Restorations suitable for repairs (composite restorations) |
| Zahnarztpraxis Dr. Portmann | zahnarztportmann.ch | CH | Heimberg (rural) | Single practitioner (male) | SSO | 1990 | - Advantages of repair restorations (preservation of tooth substance, reduced costs) |
| Zahnarztpraxis Dr. Weber | weber-zahnarzt.ch/aesthetik/zahnkorrektur/veneers-kosten/ | CH | Schindellegi (rural) | Single practitioner (male) | SSO | 1998 | - Restorations suitable for repairs (composite restorations) - Defects suitable for repairs (marginal discoloration) |
| Zahnarztpraxis Schweiger | zahnarzt-schweiger.ch/zahnmedizin-2/ | CH | Bonstetten (rural) | Multiple dentists (two female dentists) | SSO*** | 2005 [17]* | - Restorations suitable for repairs (composite restorations) |
| Dr. Geifes | dr-geifes.at/leistungen/fuellungen | AT | Wiener Neustadt (town) | Single practitioner (male) | *no information provided* | *no information provided* | - Restorations suitable for repairs (composite restorations) |
| Dr. Wiederin | zahnarzt-rheintal.at/leistungen | AT | Höchst (rural) | Single practitioner (male) | DGI, ÖGP, ÖGZMK | 1982 | - Restorations suitable for repairs (composite restorations) |
| Ordination Dr. Lemach, DDr. Hiller-Lemach | lemach.at/de/ästhetik/zahnfarbene-füllungen/ | AT | Salzburg (city) | Multiple dentists (one female, one male dentist) | *no information provided* | 1977, 2006 | - Restorations suitable for repairs (composite restorations) |
| Ordination Dr. Menapace | zahnarzt-menapace.at/de/faq/detail.asp?id=14&tit=Fuellungsmaterialien%20im%20Vergleich | AT | Salzburg (city) | Single practitioner (male) | *no information provided* | 1987 | *none* |
| Korident Zahnarztpraxis, Dr. Rita | zahnarztsopron.net/zahnarzt-sopron/asthetische-zahnfullungen | HU | Sopron (town) | Multiple dentists (one female, one male dentist) | *no information provided* | 1990, *unknown* | - Restorations suitable for repairs (composite inlays) |

Websites with information on repair restorations / restoration repair as well as practice-specific parameters: Practice location (rural / towns / cities with at least 100,000 inhabitants), practice setting (single practitioner / multiple dentists), society membership, year of examination / year in which license to practice dentistry (“Approbation”) was received, and provided information regarding repairs. *Acording to public profile at linkedin.com or xing.com. **According to CV in published dissertation. ***According to the member information of DGMZK (https://www.zahnmedizinische-patienteninformationen.de/zahnarztsuche) or SSO (https://www.sso.ch/sso/zahnarztsuche.html).

**References for the appendix**
